# Perception of Filtered Speech by Children with Developmental Dyslexia and Children with Specific Language Impairments

**DOI:** 10.3389/fpsyg.2016.00791

**Published:** 2016-05-30

**Authors:** Usha Goswami, Ruth Cumming, Maria Chait, Martina Huss, Natasha Mead, Angela M. Wilson, Lisa Barnes, Tim Fosker

**Affiliations:** ^1^Centre for Neuroscience in Education, Department of Psychology, University of CambridgeCambridge, UK; ^2^Ear Institute, University College LondonLondon, UK; ^3^School of Psychology, Queen’s University BelfastBelfast, UK

**Keywords:** temporal modulation, speech perception, phonology, dyslexia, SLI

## Abstract

Here we use two filtered speech tasks to investigate children’s processing of slow (<4 Hz) versus faster (∼33 Hz) temporal modulations in speech. We compare groups of children with either developmental dyslexia (Experiment 1) or speech and language impairments (SLIs, Experiment 2) to groups of typically-developing (TD) children age-matched to each disorder group. Ten nursery rhymes were filtered so that their modulation frequencies were either low-pass filtered (<4 Hz) or band-pass filtered (22 – 40 Hz). Recognition of the filtered nursery rhymes was tested in a picture recognition multiple choice paradigm. Children with dyslexia aged 10 years showed equivalent recognition overall to TD controls for both the low-pass and band-pass filtered stimuli, but showed significantly impaired acoustic learning during the experiment from low-pass filtered targets. Children with oral SLIs aged 9 years showed significantly poorer recognition of band pass filtered targets compared to their TD controls, and showed comparable acoustic learning effects to TD children during the experiment. The SLI samples were also divided into children with and without phonological difficulties. The children with both SLI and phonological difficulties were impaired in recognizing both kinds of filtered speech. These data are suggestive of impaired temporal sampling of the speech signal at different modulation rates by children with different kinds of developmental language disorder. Both SLI and dyslexic samples showed impaired discrimination of amplitude rise times. Implications of these findings for a temporal sampling framework for understanding developmental language disorders are discussed.

## Introduction

The proposal that human speech perception relies on multi-time resolution processing is increasingly well-supported by both behavioral and neuroimaging data (Poeppel, 2003; Greenberg, 2006; Hickok and Poeppel, 2007; Luo and Poeppel, 2007; Giraud et al., 2008; Ghitza and Greenberg, 2009; Chait et al., 2015). According to multi-time resolution models, the brain tracks the temporal modulation patterns in speech at different timescales simultaneously, via phase-locking of intrinsic cortical oscillations to modulations at corresponding timescales in the signal (Ghitza, 2011; Giraud and Poeppel, 2012; Poeppel, 2014). Endogenous neuronal oscillations in frequency bands at ‘privileged’ rates for speech (*delta*, 1 – 3 Hz, *theta*, 4 – 8 Hz, *beta*, 15 – 30 Hz, and *low gamma*, 30 – 50 Hz; rates from Poeppel, 2014) appear to provide a basis for parsing the continuous signal into linguistically relevant units (e.g., *delta* – syllable stress patterns, *theta* – syllables, *beta* – onset-rime units, *low gamma* – phonetic information, see Ghitza et al., 2012; Poeppel, 2014; Leong and Goswami, 2015). The information associated with the different timescales is then bound together to give the final speech percept. Accurate oscillatory phase locking is mediated in part by amplitude ‘rise times,’ auditory ‘edges’ associated with amplitude (energy) modulations in the continuous signal that help to specify temporal modulation rates (Gross et al., 2013; Doelling et al., 2014). Rise times appear to *phase re-set* neuronal activity, enabling accurate ‘sampling’ of the speech input in different temporal integration windows simultaneously, thereby supporting the parsing and encoding/decoding of speech (Luo and Poeppel, 2007; Poeppel, 2014).

Logically, impairments in this simultaneous ‘sampling’ of the speech signal at one or more temporal rates could be a causal factor in developmental disorders of language learning. Atypical neural sampling at one or more of the ‘privileged’ temporal rates for speech would result in subtly different acoustic information being bound together to yield the final speech percept. Such a model is proposed by the neural ‘temporal sampling’ framework for understanding developmental language disorders (TSF, Goswami, 2011). The TSF proposed that the sensory impairments in discriminating amplitude envelope (AE) rise times found in children with developmental language disorders could affect the efficiency of neuronal phase-resetting and the accuracy of neuronal entrainment to the energy patterns in speech. This would result in atypical perceptual representations, which would affect phonological development (as in developmental dyslexia), and possibly also syntactic and grammatical development (as in oral speech and language disorders). Psychoacoustic studies of children with language learning disorders consistently find impaired discrimination of non-speech AE rise times, for children with both developmental dyslexia (disordered acquisition of written language) and children with oral SLIs (disordered comprehension and production of oral language). For developmental dyslexia, studies in a range of languages (English, French, Spanish, Chinese, Dutch, Finnish and Hungarian, see Goswami, 2015 for a review of sensory data) have shown that impaired discrimination of AE rise time is related to impairments in phonological processing at multiple linguistic levels (stressed syllable, syllable, onset-rime, Chinese tone, phoneme, “rise time theory,” see Goswami, 2015). For SLI, psychoacoustic studies have so far focused on English-speaking children (Corriveau et al., 2007; Fraser et al., 2010; Beattie and Manis, 2012; Cumming et al., 2015a; Richards and Goswami, 2015). In English-speaking children with SLIs, AE rise time impairments are consistently associated with phonological impairments, and are less consistently associated with receptive and expressive language impairments.

Importantly for the neural temporal sampling approach, there is fierce debate in the developmental literature concerning whether these two developmental disorders of language learning (developmental dyslexia and oral SLIs) lie on a continuum or are distinct disorders with differing etiology. For example, an influential literature review argued that despite the many behavioral similarities in children with the two disorders, SLI and dyslexia were best conceptualized as independent developmental syndromes (Bishop and Snowling, 2004). Bishop and Snowling (2004) argued that classic SLI and classic dyslexia were different in their characteristics. Classically dyslexia is always associated with oral phonological processing impairments, while processing of the semantic and syntactic aspects of oral language are typically preserved. Conversely, classically SLI is always associated with non-phonological language impairments (e.g., in the production and comprehension of spoken language), but is not consistently associated with phonological impairments. Bishop and Snowling (2004) emphasized that developmental disorders that appear similar at the behavioral level may have different causal origins and may require different remediation.

At the same time, some studies demonstrate overlap of over 50% in the reading and language scores of children diagnosed with either SLI or dyslexia (e.g., McArthur et al., 2000). McArthur et al. (2000) and her colleagues reported that in a sample of 110 children with language impairments, 55% of those with a diagnosis of dyslexia also had oral language difficulties, while 51% of those with a diagnosis of SLI also had reading impairments. Given that both disorders (developmental dyslexia and SLI) show high heritability, any shared causal origins seem most likely to be linked to *universal* features of linguistic processing, such as the neural tracking of the different temporal modulation patterns in the speech envelope revealed by multi-time resolution models (Poeppel, 2014). Temporal sampling theory (Goswami, 2011, 2015) has highlighted the prosodic and syllable-level perceptual difficulties found in children with SLI and developmental dyslexia, theoretically associated with processing slower temporal modulations. Children with developmental dyslexia show perceptual difficulties in discriminating amplitude modulation (AM) and frequency modulation (FM) across languages, with some studies suggestive of more marked deficits at slower rates (e.g., 4 Hz AM, French, Lorenzi et al., 2000; 2 Hz FM; English, Witton et al., 1998; 2 Hz FM, Norwegian, Talcott et al., 2003). English children with dyslexia also show impaired neuronal oscillatory entrainment to rhythmic speech presented at a 2 Hz (delta band) rate (Power et al., 2013). Meanwhile, prosodic difficulties can be identified in individuals with dyslexia across languages (English: Goswami et al., 2010, 2013; Spanish: Jiminez-Fernandez et al., 2014; French: Soroli et al., 2010). A sensory/neural difficulty in developmental dyslexia centered around slow temporal modulations is thus reasonably well-supported.

Auditory studies of SLI have focused on a theory proposed by Tallal and Piercy (1973), which argued for difficulties in processing rapidly arriving acoustic information. Tallal’s ‘rapid auditory processing’ hypothesis (Tallal, 1980) was based on the finding that children with SLI were worse than TD controls in processing the temporal order of sounds when the sounds were brief (75 ms) with short (e.g., 8, 15, 30, 60 ms) interstimulus intervals (ISIs, Tallal and Piercy, 1973). The children with SLI did not differ from TD controls when ISIs were longer than 150 ms. The RAP hypothesis proposed that as the timeframe of 75 ms corresponds to the average duration of individual phonemes, a RAP deficit caused poor phonological representation of phonemes and therefore subsequent language difficulties for affected children. RAP theory has been the subject of much debate since its proposal, with some studies replicating its findings in children with SLIs (e.g., Frumkin and Rapin, 1980), and others failing to find a RAP deficit in these children (e.g., Bishop et al., 1999). Nevertheless, from the perspective of temporal sampling of the speech signal, it is logically possible that while children with both disorders share a difficulty in processing AE rise times and the temporal modulation patterns in speech, the *rates of temporal integration* that are impaired may differ for each disorder. The processing of slower temporal rates may be the primary impairment in children with dyslexia, while the processing of faster temporal rates may be the primary impairment in children with SLIs.

To date, the available literature does not enable systematic analysis of a rate-specific temporal hypothesis across developmental language disorders. Accordingly, here we investigate directly the possibility that there is a perceptual difficulty at different temporal rates in the discrimination of the modulation patterns in speech, comparing children with dyslexia to children with oral SLIs. We investigate rate-specific processing by using a novel speech filtering technique reported in a recent psychophysical study of adult speech processing by Chait et al. (2015). Chait et al. (2015) created this new filtering method in order to enable the independent estimation of the contributions made to speech perception by faster versus slower temporal modulation patterns in the signal. Their filtering method selectively extracted slow temporal envelope modulations (∼4 Hz), corresponding to the duration of syllables (∼250 ms), or faster temporal envelope modulations (∼33 Hz), corresponding to phonetic properties in speech (∼30 ms). Chait et al. (2015) then compared perceptual sensitivity to the two modulation rates in a speech intelligibility task with neurotypical adults who spoke American English. Chait et al. (2015) used target sentences which were low in contextual cues, with low transitional probabilities between words, but which were meaningful and syntactically appropriate (e.g., “The ripe taste of cheese improves with age”). They reported that their adult participants showed relatively poor recognition for the two kinds of filtered speech when presented separately (slow modulations, 42% correct; fast modulations, 19% correct). Adults showed significantly greater recognition when one type of signal was presented to each ear (slow + fast, 64% correct), suggestive of *temporal integration* (binding of the perceptual information yielded by the slow and faster modulations). Chait et al. (2015) concluded that listeners use both slow and fast modulation information for speech processing, supporting multi-time resolution models of speech perception (Poeppel, 2003; Poeppel et al., 2008).

**FIGURE 1:**
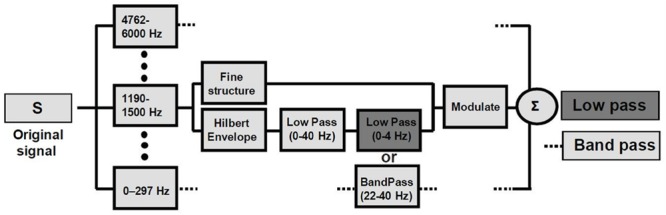
**Signal processing block diagram.** Signals were low-pass filtered at 6 kHz and sampled at 16 kHz. The frequency spectrum of the speech signal was partitioned into 14 frequency bands with a linear-phase FIR filter bank (slopes 60 dB/100 Hz or greater), spanning the range 0.1 and 6 kHz, spaced in 1/3 octave steps (approximately critical band–wide) across the acoustic spectrum. The Hilbert transform was used to decompose the signal in each band into a slowly varying temporal envelope and a rapidly varying fine structure. The temporal envelope was subsequently low-pass filtered with a cutoff frequency of 40 Hz and then either low- (0–4 Hz; dark gray bar) or band- (22–40 Hz; light gray bar) pass filtered. The time delays, relative to the original signal, introduced by the filtering, were compensated by shifting the filter outputs. After the filtering, the envelope was combined with the carrier signal (fine structure) by multiplying the original band by the ratio between the filtered and original envelopes. The result for each original signal (S) are ‘Low Pass’ and ‘Band Pass’ conditions containing predominantly low or high modulation frequencies, respectively.

In the current study, we applied the same modulation extraction technique to children’s nursery rhymes spoken in British English. We chose nursery rhymes rather than unpredictable sentences in order to make the task more accessible to our child participants. The opening lines of 10 different nursery rhymes were either low pass filtered or band pass filtered using the methods from Chait et al. (2015, see **Figure 1**), and were presented for recognition by children with and without either developmental dyslexia (Experiment 1) or speech and language impairments (SLIs; Experiment 2). In Experiment 1, each block of 10 nursery rhymes was presented four times over the course of the experiment (see Methods). When speech is degraded (e.g., by time compression or by vocoding), adults show rapid improvement in recognition during short time periods (e.g., 10–15 min, Davis et al., 2005). It was thus deemed important to look at performance as a function of presentation block (four blocks). All children were expected to improve in performance over the course of the experiment (which lasted around 30 min), but on the TSF (Goswami, 2011) children with dyslexia were expected to show selective difficulty with low pass filtered nursery rhymes, at least in the first presentation block. Impaired perception of low pass filtered speech would be indicated by an interaction between group and filter, or between, group, filter and block. For the children with SLIs, who were younger, only two blocks of sentences were presented. This took around 25 min. Again, learning was expected to occur over the course of the experiment. Of interest was whether we would find differential SLI performance with low pass versus band pass filtered speech, with potentially greater perceptual impairments for the faster modulations (Tallal and Piercy, 1973).

## Experiment 1

### Method

#### Participants

Sixty-seven children participated in Experiment 1, all of whom were taking part in an ongoing longitudinal study of auditory processing in dyslexia (see Goswami et al., 2011, 2013). All participants and their guardians gave informed consent, and the study was approved by the Psychology Research Ethics Committee of the University of Cambridge. Forty-three of the children in the study had either been identified as having developmental dyslexia by their local education authority, and/or showed severe literacy and phonological deficits according to our own test battery. The current task was administered during the third year of the study, when all children were very familiar with the experimenters and with doing auditory tasks on computers. Twenty-four age-matched control children (TD control group) were also tested. Only children who had no additional learning difficulties (e.g., dyspraxia, ADHD, autistic spectrum disorder, SLI) and non-verbal IQ within the normal range were included. All participants received a short hearing screen using an audiometer. Sounds were presented in both the left or right ear at a range of frequencies (250, 500, 1000, 2000, 4000, 8000 Hz), and all subjects were sensitive to sounds within the 20 dB HL range. At the current test point, the children with dyslexia had a significant auditory deficit in rise time perception compared to their age matched controls (mean threshold in comparison to a 15 ms standard = 127.4 ms, SD 96.8 ms). Hence on average these children could distinguish a 15 ms rise time from a 142 ms rise time. By comparison, the rise time threshold for the TD control group was 65.1 ms (SD 47.7), a mean performance level that was significantly more sensitive than that of the dyslexics (*t*[1,66] = 3.1, *p* < 0.001). Hence TD controls could on average distinguish a 15 ms rise time from a rise time of 80 ms. Other participant details are in **Table 1**.

**Table 1:** Participant details for Experiment 1.

	Dyslexic	TD Controls	*F*(1,66)
Chronological age (months)	125.7	124.4	0.17
(*SD*)	(13.1)	(12.1)	
Reading (SS)^a,b^	83.9	109.0	93.8^∗∗∗^
(*SD*)	(10.6)	(9.6)	
Spelling (SS)^a,b^	80.6	104.4	112.5^∗∗∗^
(*SD*)	(8.7)	(9.2)	
Vocabulary (SS)^b^	104.6	107.4	0.91
(*SD*)	(11.6)	(11.5)	
WISC short-form I.Q.^b^	105.4	108.6	1.0
(*SD*)	(15.0)	(10.6)	
Phonological awareness^a^	11.8	15.9	25.4^∗∗∗^
(*SD*)	(3.3)	(2.9)	
RAN in seconds^a^	39.1	34.3	6.4^∗∗^
(*SD*)	(8.6)	(4.9)	
PSTM^a^	42.0	47.6	9.2^∗∗^
(*SD*)	(6.8)	(8.3)	

*^a^DYS worse than TD; ^b^Standard Score = 100, *SD* = 15. SS, standard score; WISC, Wechsler Intelligence Scales for Children; RAN, rapid automatized naming; PSTM, phonological short-term memory. ^∗∗^*p* < 0.01, ^∗∗∗^*p* < 0.001.*

##### Procedures

Children were given standardized reading and I.Q. tests, experimental phonological awareness tasks to assure their dyslexic status (see below), and the speech recognition task based on nursery rhymes (described fully below).

##### Tasks

*Standardized reading, vocabulary and I.Q. tests.* These comprised the British Ability Scales single word reading test for English (BAS, Elliott et al., 1996), the British Picture Vocabulary Scales (Dunn et al., 1982) and four subtests of the standardized form of the Wechsler Intelligence Scale for Children (WISC; Wechsler, 1992): block design, picture arrangement, similarities, and vocabulary. Full-scale IQ scores were prorated following the procedure adopted by Sattler (1982).

##### Phonological tasks and auditory tasks.

(i) *Phonological awareness.* A rhyme oddity task using digitized speech was used (e.g., kick, pick, *tip*, see description in Thomson and Goswami, 2008). The maximum score was 20.
(ii) *Rapid automatised naming (RAN).* Two experimental RAN lists were used based on familiar objects whose names occupied either dense or sparse phonological neighborhoods (see Kuppen et al., 2011, for stimuli). Children were first introduced to the names of the pictures and then shown a page with the same pictures repeated 40 times in random order. The children were asked to produce the names as quickly as possible and were timed for each list. A rapid naming score was derived by averaging performance across the two lists.
(iii) *Phonological short-term memory (PSTM).* The memory task was also based on digitized speech, and consisted of 16 trials of four spoken monosyllables. The children were required to listen to each set of four words and then to repeat them back to the experimenter. Performance was scored by word, so the maximum score was 64. The stimulus list is available in Kuppen et al. (2011).
(iv) *Amplitude rise time (1 rise).* The rise time task (also called the 1 Rise task in our prior work) was a psychoacoustic computerized task presented in AXB format. The program used an adaptive procedure to staircase through the stimuli on the basis of the participant’s previous answer. The threshold score was derived from the mean of the last four reversals and the maximum number of trials was 40. Each trial consisted of three 800 ms tones, separated by 500 ms ISIs. Two were standard tones with a 15 ms linear rise time envelope, 735 ms steady portion and a 50 ms linear fall time. For the third tone, the onset rise time varied. The longest rise time was 300 ms. The computer screen showed the child three cartoon dinosaurs. Children were told that each dinosaur would make a sound and that their task was to decide which sound was different. It was explained that the different sound would have a softer rising sound (this equated to a longer rise time). Sound X was always the standard tone, the ‘different’ tone was either A or B. Children were able to point, verbalize or use the computer mouse to indicate their response. Feedback was given automatically by the program after each trial. Five practice trials were given before the experimental trials. The AXB format was chosen in order to minimize the memory load of the task.

*Filtered Speech Recognition Task.* This was based on 10 familiar nursery rhymes spoken at a rate of approximately 4 syllables a second (see Leong and Goswami, 2015). All the nursery rhymes used are shown in Appendix 1. A picture was found to illustrate each nursery rhyme, and children were familiarized with the pictures. On each experimental trial (40 trials in total), the children listened to filtered speech while viewing all 10 pictures and then had to select the target. In case the task was too easy, two versions of each nursery rhyme were filtered. In one case, the real rhyme was used, and in the second case the words in the opening lines of each rhyme were changed to different words or non-words preserving syllable stress and prosodic cues (e.g., *Twinkle Twinkle Little Star* became “jingle jingle riddle car”; *Incy Wincy Spider* became “izzy whizzy glider,” see Appendix). Both versions of the task were described as being spoken by a little alien who didn’t really know nursery rhymes (a cartoon icon) and who was sometimes tricky because he said the wrong words. This created a game for administering the task. The child had to decide which nursery rhyme the alien was trying to say, and then whether he was being “tricky” (i.e., whether he had got the phonetic content of the words right or not).

On each trial, the child would thus hear the beginning of a nursery rhyme or a wrongly-worded prosodically equivalent sentence whose modulation frequency was either low-pass filtered (<4 Hz) or band-pass filtered (22 – 40 Hz) in the modulation domain. Filter parameters were chosen to encompass the modulation frequencies shown to be most relevant for speech in adult studies: 4 Hz (∼250-ms-sized temporal windows) in the low pass condition and 33 Hz (∼30 ms temporal windows) in the band pass condition. These values were further motivated by the pervasive relevance of these time ranges in speech, non-speech and brain-imaging studies (see Zatorre and Belin, 2001; Poeppel, 2003; Boemio et al., 2005; Hesling et al., 2005; Santoro et al., 2014; and references therein). In Chait et al. (2015), the interaction between the different types of information was of critical interest, and so the two conditions were separated as much as possible in modulation-frequency space (see **Figure 1**). This separation comes at the cost of significant information reduction in the signal and consequently a decline in intelligibility. Nevertheless, these two conditions are of interest with respect to temporal sampling theory in developmental dyslexia (Goswami, 2011). Temporal sampling theory proposes a specific difficulty with slow temporal modulations in the theta and delta band frequency ranges. If this proposal is correct, we should find significantly poorer performance for the children with dyslexia compared to TD controls for the low pass filtered stimuli only.

For each filtered stimulus, the child had to decide which nursery rhyme the alien was trying to say by selecting a target picture (one of 10). The rhyme was either the filtered version of the standard words (low pass or band pass) or the filtered version of the wrong words (low pass or band pass). Each nursery rhyme (standard words, wrong words) was given once as low pass filtered speech and once as band pass filtered speech, in semi-random order, so that each nursery rhyme (standard words or wrong words) occurred once in each of the four blocks. Presentation was continuous, so that the child was not aware of the “blocks,” however, the children perceived the task to be difficult and said it was tiring. Therefore, if a short break was requested, this was given after completion of the first 20 trials (i.e., after Block 2 and before Block 3). About a third of the sample (13 dyslexic, 10 TD control) requested a break, which was usually spent doing a different task that is not part of the current report. On each trial, the child was also asked whether the alien had been tricky and said the wrong words.

The signal processing was carried out by the last author, using Matlab code supplied by the third author. The code was created using the signal processing procedure shown in **Figure 1**, which is an extension of the method used by Drullman et al. (1994a,b). The stimuli were created off-line and saved in stereo WAV format at a sampling rate of 16 kHz. They were presented over high-quality headphones (Sennheiser HD580) at a comfortable listening level. Responses were scored in terms of recognition of the target nursery rhyme by choosing the correct picture. The design was fully counterbalanced and is shown in Appendix 1.

### Results

Recognition data in each of the four blocks indicating whether the correct target picture was selected are presented as **Table 2**. These data indicate whether the filtered speech was recognizable as a particular nursery rhyme. It can be seen that both the children with dyslexia and the control children found the low pass filtered rhymes quite easy to recognize (76 and 78% correct respectively on Block 1). In contrast, the band pass filtered rhymes were more difficult for both groups, although recognition was significantly above chance in each case [Block 1 data, 31% for dyslexics, *t*(42) = 6.8, *p* < 0.0001; 33% for TD, *t*(23) = 6.6, *p* < 0.0001]. There are no obvious group differences in speech recognition, however, there are clear learning effects during the experiment. These appear to be strongest for the more difficult band pass filtered rhymes. Performance with the band pass filtered rhymes improves by 31% for the children with dyslexia across the four presentation blocks, and by 38% for the control children. The only apparent difference between groups is more rapid learning by the control children, who make 32% of their improvement on the band pass filtered rhymes in the second presentation block. In this block, the target rhymes had previously been heard as low pass filtered speech, suggesting more acoustic learning from low pass filtered targets in the TD children than in the children with dyslexia.

**Table 2:** Recognition accuracy (% target rhymes identified) by presentation block, with standard errors in parentheses.

Step	Block 1	Block 2	Block 3	Block 4	Total
**Low pass, <4 Hz**					
DYS	76 (2.9)	89 (2.7)	84 (2.3)	91 (2.2)	85
TD	78 (3.8)	92 (3.6)	90 (3.1)	97 (2.9)	89
Total low pass	77	91	87	94	
**Band pass, 22 – 40 Hz**					
DYS	31 (4.1)	43 (4.3)	50 (4.1)	62 (4.0)	47
TD	33 (5.4)	65 (5.7)	62 (5.5)	71 (5.4)	58
Total band pass	32	54	56	67	

To explore these different effects statistically, we first computed an omnibus analysis using all blocks. As this omnibus analysis combines learning effects due to task practice and due to acoustic learning from previously hearing either the low pass or band pass filtered forms of the target rhymes, we then explored specific learning effects from low versus band pass filtered speech by analyzing data from Blocks 1 and 2 only. In Block 2, the entire band pass targets were previously heard as low pass filtered speech, and the entire low pass targets were previously heard as band pass filtered speech. Hence improvement in perceiving band pass filtered speech from Block 1 to Block 2 would reflect acoustic learning from low pass filtered speech, while improvement in perceiving low pass filtered speech from Block 1 to Block 2 would reflect acoustic learning from band pass filtered speech. When comparing Block 1 versus Block 2 data only, a significant interaction between Group and Filter would suggest differential perceptual recognition effects, whereas a significant interaction between Block, Group and Filter would show differential learning by group from the different filters used.

The omnibus 2 × 2 × 4 (Group [Dyslexic, TD] × Filter [low pass, band pass] × Block [1, 2, 3, 4]) ANOVA used the number of target pictures selected correctly as the dependent variable. Newman–Keuls *post hoc* tests were used to inspect significant effects. The ANOVA showed significant main effects of Filter, *F*(1,65) = 372.5, *p* < 0.0001, ηρ^2^ = 0.791, because performance was better with low pass filtered speech, Block, *F*(3,195) = 28.7, *p* < 0.0001, ηρ^2^ = 0.306, because performance improved from Block 1 to Block 2, and from Block 3 to Block 4, and Group, *F*(1,65) = 5.8, *p* < 0.05, ηρ^2^ = 0.082, because over the experiment as a whole the control children performed significantly better than the children with dyslexia. There was also a significant interaction between Block × Filter, *F*(3,195) = 5.3, *p* < 0.01, ηρ^2^ = 0.075, however, the interaction between Group × Filter did not approach significance, *F*(1,65) = 1.7. The interaction between Block and Filter arose because for the low pass filtered speech, the only significant improvement was from Block 1 to Block 2, whereas for the band pass filtered speech, improvements from Block 1 to Block 2, and from Block 3 to Block 4 were both significant. The small reductions in performance between Blocks 2 and 3 visible in **Table 2** were non-significant, and may reflect the short rest taken by some children between Blocks 2 and 3.

To assess the possibility of specific group differences in learning from the two kinds of filtered speech, we analyzed recognition of low pass filtered versus band pass filtered speech on the first versus second occasion that each type of speech was heard (Blocks 1 and 2 data only) using a 2 × 2 × 2 (Group [Dyslexic, TD] × Filter [low pass, band pass] × Block [1, 2]) ANOVA. Again, the number of target pictures selected correctly was the dependent variable. This second analysis enabled comparison of improvements in nursery rhyme recognition after the target rhyme had previously been heard once through the opposite filter. This enabled us to test learning from previously hearing the target rhyme as low pass filtered speech, now heard again as a band pass filtered target on Block 2, and to compare this with learning from previously hearing the target rhyme as band pass filtered speech, now heard again as a low pass filtered target on Block 2. The ANOVA showed significant main effects of Block, *F*(1,65) = 57.4, *p* < 0.0001, ηρ^2^ = 0.469, and Filter, *F*(1,65) = 192.4, *p* < 0.0001, ηρ^2^ = 0.747, and significant interactions between Group × Block, *F*(1,65) = 6.7, *p* < 0.01, ηρ^2^ = 0.093, and Group × Block × Filter, *F*(1,65) = 4.2, *p* < 0.05, ηρ^2^ = 0.061. The interaction between Group × Filter did not approach significance, *F*(1,65) = 1.9. *Post hoc* tests (Newman–Keuls) of the three-way interaction showed that while both groups showed equivalent (13% versus 14%) learning effects from hearing band pass filtered speech, the children with dyslexia showed significantly poorer learning from hearing low pass filtered speech (12%) compared to the typically developing (TD) children (32%, *p* < 0.01). Hence the children with dyslexia benefitted less than TD controls from previously hearing the target spoken as low pass filtered speech, but showed similar benefits to TD controls for band pass filtered speech. The *perceptual learning effects* from hearing low pass filtered speech by group were significantly different.

Finally, we analyzed whether the children could hear whether the tricky alien was saying the words in the nursery rhymes correctly or not (see **Table 3**). This analysis was expected to provide a measure of children’s sensitivity to phonetic rather than prosodic information in the filtered speech. A second 2 × 2 × 4 (Group [Dyslexic, TD] × Filter [low pass, band pass] × Block [1, 2, 3, 4]) omnibus ANOVA was run, taking the number of trials in which the child identified the correct target picture and also decided correctly whether the tricky alien had said the right words or not as the dependent variable. Performance was significantly above chance in all conditions, even for those conditions showing low accuracy [e.g., dyslexics = 16% correct for band pass filtered speech in Block 1, *t*(42) = 3.9, *p* < 0.001; controls = 23% correct for band pass filtered speech in Block 1, *t*(23) = 5.0, *p* < 0.001]. The ANOVA showed significant main effects of Filter, *F*(1,65) = 255.6, *p* < 0.0001, ηρ^2^ = 0.797, because performance was better with low pass filtered speech, and Block, *F*(3,195) = 21.8, *p* < 0.0001, ηρ^2^ = 0.251, because performance improved from Block 1 to Block 2, and from Block 2 to Block 3, but not from Block 3 to Block 4. There was no significant main effect of Group, *F*(1,65) = 1.4. The interaction between Block × Filter, *F*(3,195) = 4.6, *p* < 0.01, ηρ^2^ = 0.066, was the only significant interaction. It arose because whereas significant improvements for low pass filtered stimuli occurred from Block 1 to Block 2 only, significant improvements for band pass filtered speech occurred both from Block 1 to Block 2, and from Block 2 to Block 3. The lack of significant group effects or interactions by group suggest that the children with dyslexia were not impaired at recovering phonetic-level information from the filtered stimuli. The finding that both groups of children found it easier to recover information from low pass filtered speech supports the importance of slower temporal modulations in the AE in speech recognition, even when the information to be recovered is phonetic rather than prosodic or syllabic.

**Table 3:** Speech recognition accuracy (% target rhymes correctly identified as trick or real) by presentation block, with standard errors in parentheses.

Step	Block 1	Block 2	Block 3	Block 4	Total
**Low pass, <4 Hz**					
DYS	62 (2.6)	66 (3.6)	71 (3.0)	72 (3.1)	68
TD	62 (3.4)	70 (4.7)	77 (3.9)	72 (4.1)	70
Total low pass	62	68	74	72	
**Band pass, 22 – 40 Hz**					
DYS	16 (3.4)	29 (4.2)	39 (3.9)	43 (3.7)	32
TD	23 (4.5)	37 (5.5)	42 (5.1)	50 (4.8)	38
Total band pass	20	33	41	47	

In order to assess the relationships between children’s sensory perception and their performance in the vocabulary, reading and phonological tasks, we computed partial correlations between the measures of filtered speech perception, learning from filtered speech and rise time discrimination and the different outcome measures, partialling out age and non-verbal IQ. The correlations are shown in **Table 4**. As can be seen by inspecting the table, both measures of filtered speech recognition and the learning measure for low pass filtered speech showed significant correlations with reading and spelling. As would be expected on the basis of prior work utilizing the TSF, individual differences in sensitivity to AE rise time were significantly related to the phonological, reading and spelling measures (e.g., Richardson et al., 2004; Huss et al., 2011), with the exception here of RAN (*r* = 0.18). A series of three-step fixed entry multiple regression equations were also computed (entering recognizing low pass filtered speech, recognizing band pass filtered speech, learning from low pass filtered speech, and learning from band pass filtered speech respectively at step 3), to assess whether the filtered speech measures accounted for significant unique variance in the outcome measures in our sample of 68 children (phonological awareness, phonological memory, RAN, reading, spelling and BPVS vocabulary, hence 24 equations in all). In each equation we entered age at step 1 and non-verbal IQ at step 2, and then the different perceptual variables respectively at step 3. As might be expected from **Table 4**, individual differences in both recognizing low pass filtered speech and learning from low pass filtered speech accounted for significant independent variance in children’s reading and spelling development (reading: recognize low pass = 12%, β = 0.35, *t* = 3.1, *p* = 0.003; learn from low pass = 13%, β = 0.37, *t* = 3.1, *p* = 0.003; spelling: recognize low pass = 17%, β = 0.41, *t* = 3.6, *p* = 0.001; learn from low pass = 12%, β = 0.35, *t* = 2.9, *p* = 0.005). Individual differences in the recognition of band pass filtered speech predicted significant unique variance in more of the outcome measures: phonology (7%, β = 0.27, *t* = 2.2, *p* < 0.05), RAN (10%, β = -0.34, *t* = 2.9, *p* = 0.005), reading (14%, β = 0.39, *t* = 3.2, *p* = 0.002), spelling (16%, β = 0.42, *t* = 3.5, *p* = 0.001) and approached significance for vocabulary development (4%, β = 0.22, *t* = 2.0, *p* = 0.054). Learning from band pass filtered speech predicted significant unique variance in one outcome measure only, RAN (13%, β = 0.24, *t* = 2.0, *p* = 0.045), and showed a positive relationship, suggesting paradoxically that children who showed greater learning from band pass filtered speech also had poorer rapid naming skills. Overall, the regression equations suggest that the ability to recognize speech information on the basis of *both* slower and faster temporal modulations is related to the development of *both* spoken and written language skills. Notably, none of the outcome measures were related to phonological memory, suggesting that the filtered speech tasks are tapping into basic perceptual processes.

**Table 4:** Partial correlations between children’s performance in the phonology, vocabulary and reading/spelling outcome measures and their filtered speech performance and rise time discrimination thresholds, controlling for age and IQ.

	Recognize LP	Recognize BP	Learn from LP	Learn from BP	Rise time threshold
Phonology (Oddity task)	0.17	0.27^∗^	0.18	0.06	**-0.40^∗∗∗^**
PSTM	0.10	0.11	**-**0.06	0.02	**-0.39^∗∗∗^**
RAN	**-**0.17	**-**0.35^∗∗^	**-**0.13	0.25^∗^	0.18
BAS reading SS	0.36^∗∗^	0.38^∗∗^	0.37^∗∗^	0.01	**-0.39^∗∗∗^**
BAS Spelling SS	**0.41^∗∗∗^**	**0.40^∗∗∗^**	0.34^∗∗^	0.01	**-**0.30^∗^
BPVS SS	0.11	0.24^∗^	0.11	0.09	**-**0.27^∗^

*^∗^*p* < 0.05, ^∗∗^*p* < 0.01, ^∗∗∗^*p* < 0.001. LP, low pass filtered speech; BP, band pass filtered speech, PSTM, phonological short-term memory, RAN, rapid automatized naming; BAS, British Ability Scales; SS, standard score; BPVS, British Picture Vocabulary Scales. Uncorrected significance values; only the bolded values remain significant after applying the Benjamini and Hochberg (1995) correction for false discovery.*

### Discussion

Overall, contrary to what may be expected on the basis of TS theory, the nursery rhyme recognition paradigm used here did not reveal the expected poorer recognition of low pass filtered speech targets by children with dyslexia. The children with dyslexia showed good accuracy even in the first block of trials (76% correct), and performed at a similar level to the TD control children (78% correct). However, hearing target rhymes as low pass filtered speech did not lead to equivalent *perceptual learning* by the children with dyslexia, which is consistent with the TSF. The children with dyslexia improved by only 12% in the second block of trials, compared to 32% improvement for the control children. Notably, *both* groups of children showed better recognition of low pass filtered speech than of band pass filtered speech, as was found for the adults studied by Chait et al. (2015). Indeed, our child participants (>60% correct by Block 4, see **Table 2**) outperformed Chait et al.’s (2015) neurotypical adults with band pass filtered stimuli (the adults scored on average 19% correct). This difference is probably explained by our choice of familiar nursery rhymes rather than unpredictable sentences as target stimuli. If we had chosen less familiar targets, it is conceivable that the children with dyslexia studied here would also have shown recognition impairments for low pass filtered speech. Notably, the dyslexic and TD children showed *equivalent* perceptual learning from the band pass filtered stimuli (13 and 14% improvement respectively). This could suggest that children with dyslexia do not show language learning impairments when speech information is restricted to faster temporal modulations. Nevertheless, the partial correlation and regression analyses showed that individual differences in processing *both* slow and faster temporal modulations were significantly related to individual differences in the outcome measures, which assessed both spoken and written language skills.

Regarding our ‘rate-specific’ research question, it is important to note that the children with dyslexia showed equivalent accuracy to the TD children in extracting phonetic information from filtered speech in the “tricky alien” conditions (see **Table 3**). Processing efficiency was equal in the two groups for both the band pass filtered targets (32% correct for children with dyslexia, 38% correct for control children), and the low pass filtered targets (68% correct for children with dyslexia, 70% correct for control children). These data show that in the “tricky alien” condition, which broadly equated prosodic and syllabic structure but altered phonetic content, *both groups* of children extracted more phonetic information from the low pass filtered sentences. This may be suggestive of preserved processing of phonetic information in speech in children with developmental dyslexia, which does not support the extension of Tallal’s RAP theory to dyslexia (see Tallal, 1980, 2004). Indeed, the same dyslexic children studied here exhibited superior processing of rapid frequency information compared to the same TD children when discriminating synthetic speech syllables (Ba versus Wa, see Goswami et al., 2011). The children with dyslexia were able on average to discriminate a frequency rise of 15 ms that changed Ba to Wa, while the control children required on average a 30 ms frequency rise to make this phonetic discrimination. This could suggest that temporal integration at rapid timescales is preserved in children with dyslexia. We turn now to considering children with SLIs, the disorder originally proposed to reflect impairments in processing rapid acoustic information (Tallal and Piercy, 1973). Most recently, Tallal (2004) has argued for impairments in temporal integration windows of ∼40 ms (‘phonetic’ rate) in children with *both* developmental dyslexia and SLIs. A separate cohort study of younger children with SLIs, also ongoing in our laboratory, enabled us to administer the filtered speech tasks to children with this oral language learning disorder also.

## Experiment 2

### Method

#### Participants

Ninety-five children aged on average 9 years 6 months participated in Experiment 2, of whom 45 were referred by their schools as having a specific language impairment, which was confirmed by our own test battery. All participants and their guardians gave informed consent, and the study was approved by the Psychology Research Ethics Committee of the University of Cambridge. Only children who had no additional diagnoses of learning difficulties (e.g., dyspraxia, ADHD, autistic spectrum disorder, dyslexia) and English as the first language spoken at home were included. The absence of additional learning difficulties was based on the reports of teachers and speech and language therapists in schools, and our own testing impressions of the children. Nevertheless, our cognitive screening measures (WISC, Ravens, see Cumming et al., 2015a for detail) showed a range of standardized IQ scores in the SLI sample, from 55 to 130 (standard score = 100, *SD* = 15). This was partly due to the children’s language impairments, which impeded success on certain test items, particularly for the verbal subscales (see Cumming et al., 2015a). Hereafter we focus on non-verbal IQ for these children, and we also analyze data for sub-groupings of the SLI children with preserved non-verbal IQ but either no phonological impairments (‘classic’ SLI, Bishop and Snowling, 2004; hereafter Pure SLI) or with additional phonological impairments.

Forty-five of the children (31 male, 14 female; mean age 9 years, 6 months; range 6 years 4 months to 12 years 1 month) either had a statement of SLI from their local education authority, or had received special help for language via the teacher(s) with responsibility for special educational needs in school, and/or showed severe language deficits according to our own test battery. All children with SLI were assessed experimentally using two expressive and two receptive subtests of the Clinical Evaluation of Language Fundamentals-3 (CELF-3; Semel et al., 1995), and were included in the study if they scored at least 1 SD below the mean on two or more of these subtests. Further description of the sample, including individual CELF and NVIQ scores for each SLI child, is available in Cumming et al. (2015a).

All children received a short hearing screen using an audiometer. Sounds were presented in both the left and the right ear at a range of frequencies (250, 500, 1000, 2000, 4000, 8000 Hz), and all children were sensitive to sounds within the 20 dB HL range. The children with SLIs had a significant auditory deficit in rise time discrimination compared to their age matched controls (see also Cumming et al., 2015a). Their mean threshold in comparison to a 15 ms standard was 170.3 ms (SD 83 ms), while the mean rise time threshold for the TD control group was 108 ms (SD 80ms, (*t*[1,93] = 3.7, *p* < 0.0001). Hence on average the children with SLIs could distinguish a 15 ms rise time from a 185 ms rise time, while the TD 9-year-old controls could distinguish a 15 ms rise time from a 123 ms rise time. Other participant details are in **Table 5**.

**Table 5:** Participant details for full sample, Experiment 2.

	SLI	TD Controls	*F*(1,93)
Chronological age (months)	114.0	110.0	1.2
(*SD*)	(19.5)	(16.5)	
Reading SS^a,b^	82.8	112.5	69.4^∗∗∗^
(*SD*)	(18.9)	(15.8)	
Spelling SS	81.6	111.6	65.4^∗∗∗^
(*SD*)	(20.7)	(15.4)	
Vocabulary SS^a,b^	87.5	110.3	92.8^∗∗∗^
(*SD*)	(11.4)	(11.7)	
WISC short-form non-verbal IQ^a,b^	80.7	104.3	40.4^∗∗∗^
(*SD*)	(19.8)	(16.4)	
Phonological awareness^a^	10.3	15.8	49.9^∗∗∗^
(*SD*)	(4.0)	(3.5)	
RAN in seconds^a^	52.7	38.2	16.9^∗∗∗^
(*SD*)	(22.1)	(11.0)	
PSTM^a^	32.1	44.2	36.6^∗∗∗^
(*SD*)	(10.3)	(9.1)	

*^a^SLI worse than TD; ^b^Standard score = 100, *SD* = 15.*

#### Procedures

The children were given the same standardized reading and I.Q. tests and the same experimental phonological awareness tasks as used in Experiment 1 (see **Table 5**). They also received the same filtered speech recognition task based on 10 nursery rhymes. However, to simplify this task for the children with SLIs (who were younger, and some of whom, as noted, were of lower IQ), the “tricky” stimuli were not used. By omitting the items in which words in the opening lines of each nursery rhyme were changed to different words, as in *Twinkle Twinkle Little Star*/“jingle jingle riddle car,” we were able to present each rhyme as both low pass filtered and band pass filtered speech in two testing blocks. This meant that a rest mid-way through the experiment was not required for these younger participants, nevertheless the total testing time for the filtered speech task was approximately 25 min, similar to Experiment 1.

### Results

Data analyses utilized three groupings of the children with SLIs, to reflect the fact that phonological processing difficulties are not considered a characteristic of classic SLI (Bishop and Snowling, 2004; please see Cumming et al., 2015a, for more detail regarding these groupings). As there is no theoretical reason to expect auditory processing skills to vary with IQ (see Kuppen et al., 2011), we first analyzed data for the entire sample of SLI children, with IQ varying (**Table 5**). Data from two independent sub-groupings of SLI children with preserved IQ were also analyzed (see **Table 6** for participant details). One sub-grouping comprised SLI children with no accompanying reading or phonological difficulties, who were compared to an IQ-matched sample of 16 TD children (‘Pure SLI’ group, *N* = 16, 11 boys; for individual data on these children, please see Cumming et al., 2015a). The second sub-grouping (*N* = 15, four boys) comprised a separate sample of the SLI children, also with preserved IQ when compared to a separate matched TD sample (*N* = 15), but with reading difficulties (defined as having a SS < 85 on at least two of the standardized measures of reading and spelling used in the larger study, see Cumming et al., 2015a for further detail). These children also showed significant phonological difficulties on the experimental measures of phonological processing and are hereafter termed the ‘SLI PPR’ (poor phonology and reading) group. Note that the SLI PPR children would not qualify for a diagnosis of developmental dyslexia in the United Kingdom because of their spoken language impairments. Note further that as the TD controls for the Pure SLI grouping and the SLI PPR grouping were partly similar and partly different, we could not incorporate all three groups into one ANOVA (Pure SLI, SLI PPR, TD), as this removed the IQ-matching. As can be seen from **Table 7**, even when including the lower IQ children, children with SLIs were able to perceive the low pass filtered speech sentences very successfully, performing at 84% correct even in Block 1, and performing above chance (which would be 10%) with the band pass filtered speech also (at 29% correct, *t*[44] = 5.9, *p* < 0.0001).

**Table 6:** Participant characteristics by matched SLI sub-group.

	Pure SLI *N* = 16	Controls *N* = 16	*F*(1,31)	SLI PPR *N* = 15	Controls *N* = 15	*F*(1,29)
Age in months	109.4 (20.8)	106.6 (17.1)	0.2	115.5 (14.0)	107.6 (17.2)	1.9
WISC NVIQ SS^a^	91.1 (19.6)	96.1 (14.5)	0.7	87.8 (14.1)	95.7 (14.9)	2.2
Ravens SS^a^	95.3 (14.1)	93.8 (10.2)	0.1	83.3 (14.7)	92.3 (8.8)	4.1
Vocabulary SS^a,b^	94.2 (9.3)	104.5 (8.6)	10.5^∗∗b^	86.6 (10.3)	104.2 (8.9)	25.4^∗∗∗b^
Reading SS^a^	101.8 (12.3)	104.8 (10.5)	0.6	73.9 (9.7)	104.5 (10.8)	67.2^∗∗∗b^
Spelling SS^a^	101.2 (17.6)	106.3 (12.7)	0.9	68.4 (8.9)	105.1 (12.3)	87.6^∗∗∗b^
Phonological awareness	13.4 (4.3)	15.0 (3.1)	1.5	8.4 (3.0)	14.9 (3.1)	33.3^∗∗∗b^
PSTM (words correct)	36.3 (13.5)	41.8 (8.0)	1.9	30.4 (8.1)	42.4 (7.8)	17.1^∗∗∗b^
RAN (seconds)	45.7 (24.4)	36.4 (7.0)	2.2	55.4 (18.2)	35.7 (6.3)	15.8^∗∗∗b^

*Standard deviations in parentheses. ^∗^*p* < 0.05, ^∗∗^*p* < 0.01, ^∗∗∗^*p* < 0.001. SS, standard score; NVIQ, non-verbal IQ; PSTM, phonological short-term memory; RAN, rapid automatized naming. ^a^Standard score = 100, *SD* = 15; ^b^SLI worse than TD.*

**Table 7:** Recognition accuracy (% target rhymes identified) by presentation block in Experiment 2, with standard errors in parentheses.

	Block 1	Block 2	Total
**Low pass, <4 Hz**			
All SLI	84 (0.02)	93 (0.02)	89
All TD	92 (0.02)	96 (0.02)	94
Total	88	95	
**Band pass, 22 – 40 Hz**			
All SLI	29 (0.03)	55 (0.04)	42
All TD	48 (0.03)	70 (0.04)	59
Total	39	63	
**Low pass, <4 Hz**			
Pure SLI	89 (0.04)	96 (0.02)	93
Matched TD	96 (0.04)	99 (0.02)	97
Total	93	98	
**Band pass, 22 – 40 Hz**			
Pure SLI	28 (0.05)	60 (0.06)	44
Matched TD	45 (0.05)	74 (0.06)	60
Total	38	67	
**Low pass, <4 Hz**			
SLI PPR	83 (0.04)	87 (0.04)	85
Matched TD	97 (0.04)	99 (0.04)	98
Total	90	93	
**Band pass, 22 – 40 Hz**			
SLI PPR	24 (0.05)	55 (0.06)	40
Matched TD	44 (0.05)	73 (0.06)	59
Total	34	64	

For each grouping, we analyzed the recognition of low pass filtered versus band pass filtered speech on the first versus second occasion that each type of speech was heard (Blocks 1 and 2). We ran three separate 2 × 2 × 2 (Group [SLI, TD] × Filter [low pass, band pass] × Block [1, 2]) ANOVAs. The number of target pictures selected correctly was the dependent variable in each case. As well as analyzing overall recognition of the target nursery rhymes by filter and group, the ANOVAs enabled comparison of *improvements* in nursery rhyme recognition after the target rhyme had previously been heard once through the opposite filter. This again enabled us to assess perceptual learning effects during the course of the experiment.

The ANOVA for the full sample (45 SLI children and 50 TD controls) showed significant main effects of Block, *F*(1,93) = 88.5, *p* < 0.0001, ηρ^2^ = 0.487, and Filter, *F*(1,93) = 421.0, *p* < 0.0001, ηρ^2^ = 0.819, and significant interactions between Group × Filter, *F*(1,65) = 8.0, *p* < 0.005, ηρ^2^ = 0.079, and Block × Filter, *F*(1,93) = 21.5, *p* < 0.0001, ηρ^2^ = 0.188. The main effect of Group was also significant, *F*(1,93) = 17.2, *p* < 0.0001, ηρ^2^ = 0.156. As for the children with dyslexia, perception was significantly better for the low pass filtered speech, and performance was more accurate during the second block compared to the first block. However, the children with SLIs performed much more poorly with the band pass filtered speech compared to the TD control children. Exploration of the significant Group × Filter interaction using Newman–Keuls *post hoc* tests showed that this effect arose because while performance with low pass filtered speech was equivalent between the groups (89% for SLI, 94% for TD), performance with band pass filtered speech was significantly poorer for the children with SLIs (42% for SLI, 59% for TD, *p* < 0.001). There were no signs in the data of group differences in acoustic learning from the filtered stimuli. The degree of learning from low pass filtered speech was computed by subtracting accuracy with the band pass filtered targets in Block 1 from accuracy with the band pass filtered targets in Block 2. Learning from low pass filtered speech was 26% for the children with SLIs and 22% for the TD controls. The degree of learning from band pass filtered speech was computed by subtracting accuracy with the low pass filtered targets in Block 1 from accuracy with the low pass filtered targets in Block 2. Learning from band pass filtered speech was 9% for the children with SLIs and 4% for the TD controls. Neither group difference was significant. Hence the children with SLIs and varying IQ showed reduced recognition of band pass filtered speech targets compared to TD controls, equivalent recognition of low pass filtered speech targets, and equivalent learning effects at both modulation rates to TD children.

We next explored whether this selective difficulty in perceiving band pass filtered speech would be found for the Pure SLI sub-grouping, children who had oral speech and language difficulties but preserved IQ and no reading or phonological difficulties. The ANOVA for the Pure SLI sample (16 SLI children and 16 TD controls) showed significant main effects of Block, *F*(1,30) = 72.6, *p* < 0.0001, ηρ^2^ = 0.708, and Filter, *F*(1,30) = 215.2, *p* < 0.0001, ηρ^2^ = 0.878, and a significant interaction between Block × Filter, *F*(1,30) = 24.5, *p* < 0.0001, ηρ^2^ = 0.449. The main effect of Group was also significant, *F*(1,30) = 5.8, *p* < 0.01, ηρ^2^ = 0.161, and the Group × Filter interaction approached significance, *F*(1,30) = 3.2, *p* = 0.083, ηρ^2^ = 0.097. *Post hoc* inspection of the means in this theoretically important interaction (Newman–Keuls) showed no group difference in perceiving the low pass filtered speech, but a significant group difference in perceiving the band pass filtered speech (*p* < 0.001). This is supportive of Tallal’s proposal that having SLIs is associated with perceptual difficulties in processing faster temporal information in speech (here, AMs in the envelope at around 33 Hz, the assumed phonetic rate). The degree of learning from low pass filtered versus band pass filtered speech was again computed for each group, and again did not differ. The learning effects were 32% for low pass filtered speech for the children with SLIs compared to 29% for the TD controls, and 7% for band pass filtered speech compared to 3% for the control children.

Finally, the ANOVA for the independent SLI PPR sub-group (15 SLI children and 15 TD controls) showed significant main effects of Block, *F*(1,28) = 50.2, *p* < 0.0001, ηρ^2^ = 0.642, and Filter, *F*(1,28) = 162.5, *p* < 0.0001, ηρ^2^ = 0.853, and a significant interaction between Block × Filter, *F*(1,28) = 32.1, *p* < 0.0001, ηρ^2^ = 0.534. The main effect of Group was also significant, *F*(1,28) = 10.0, *p* < 0.005, ηρ^2^ = 0.262. However, the conceptually important Group × Filter interaction was not significant, *F*(1,28) = 0.8, *p* = 0.374, ηρ^2^ = 0.028. Therefore, the SLI PPR children showed a different pattern from the Pure SLI children in the filtered speech recognition tasks. They were significantly worse than the TD controls in perceiving filtered speech in both tasks, irrespective of whether slower or faster modulations had been extracted. Indeed, inspection of **Table 7** shows a consistent recognition deficit across the experiment for the SLI PPR group of around 10% for low pass filtered speech, and 20% for band pass filtered speech. However, there was no evidence of differential learning during the experiment compared to their TD controls. Learning effects were 31% for low pass filtered speech compared to 29% for controls, and 4% for band pass filtered speech compared to 2% for the controls. Overall, the subgroup data suggest an intriguing difference between SLI children with preserved IQ and purely oral SLIs, and SLI children with preserved IQ, oral impairments and additional phonological difficulties. Those children with SLI and preserved IQ who also had phonological difficulties showed impairments in recognizing *both* low pass filtered speech and band pass filtered speech. Children with Pure SLI showed impaired recognition of band pass filtered speech only, at least in the current experimental paradigm.

To assess the relations between the children’s sensory perception and their performance in the vocabulary, reading, phonological and language tasks, we again computed partial correlations between the different filtered speech measures and rise time discrimination, and the different outcome measures. The partial correlations are shown in **Table 8**. As can be seen by inspecting the table, the patterns are similar to those found for the children with developmental dyslexia (see **Table 4**). In particular, both measures of filtered speech recognition were correlated with individual differences in phonology. Recognition of band pass filtered speech was strongly associated with the receptive and expressive oral language measures. As in Experiment 1, a series of three-step fixed entry multiple regression equations were also computed for the sample (*N* = 95), using the full range of eight different outcome measures (phonological awareness, phonological memory, RAN, reading, spelling, BPVS vocabulary, and the CELF receptive and CELF expressive language scores) and the four different filtered speech measures (recognizing low pass filtered speech, recognizing band pass filtered speech, learning from low pass filtered speech, and learning from band pass filtered speech respectively). There were 32 equations overall, which again always entered age at step 1 and non-verbal IQ at step 2, and the different perceptual variables respectively at step 3. As might be expected from **Table 8**, the equations in which the perceptual measures accounted for significant unique variance at step 3 were largely confined to the two filtered speech recognition measures. Individual differences in the recognition of low pass filtered speech accounted for significant unique variance in phonology (9%, β = 0.30, *t* = 3.8, *p* < 0.0001), phonological short-term memory (3%, β = 0.16, *t* = 2.1, *p* = 0.041), spelling (9%, β = 0.30, *t* = 3.8, *p* = 0.011), and CELF expressive language scores (3%, β = 0.17, *t* = 2.1, *p* = 0.036). For the other outcome measures, recognition of low pass filtered speech only approached significance in each case (*p*’s < 0.07). Individual differences in the recognition of band pass filtered speech accounted for significant independent variance in almost all measures. For phonological awareness, 7% of unique variance was accounted for (β = 0.30, *t* = 3.4, *p* < 0.001), for phonological short-term memory 8% (β = 0.32, *t* = 3.9, *p* < 0.0001), for reading 5% (β = 0.26, *t* = 2.9, *p* = 0.005), for spelling 5% (β = 0.25, *t* = 2.7, *p* = 0.009), for BPVS vocabulary 5% (β = 0.26, *t* = 3.1, *p* = 0.003), for CELF receptive language scores 12% (β = 0.39, *t* = 4.8, *p* < 0.0001), and for CELF expressive language scores, 10% (β = 0.35, *t* = 4.2, *p* < 0.0001). The learning from filtered speech measures only accounted for significant unique variance in two outcome measures, RAN and BPVS, for the learning from band pass filtered speech measure only (RAN, 3%, β = -0.17, *t* = 2.0, *p* = 0.047; BPVS, 3%, β = -0.16, *t* = 2.1, *p* = 0.041). For RAN, the negative relationship indicates that children who showed more perceptual learning from band pass filtered speech also showed faster rapid naming skills. This is the opposite result to that found for children with dyslexia in Experiment 1, however, both are relatively weak effects and may be unreliable. For receptive vocabulary, the negative relationship is counter-intuitive, suggesting that children who showed more perceptual learning from band pass filtered speech also showed poorer vocabulary development. As the latter finding is contradicted by the more comprehensive CELF receptive language measures, for which recognizing band pass filtered speech accounted for 12% of unique variance, it may be unreliable. Overall, the partial correlation and regression analyses again suggest that individual differences in processing *both* slow and faster temporal modulations is significantly related to individual differences in both spoken and written language outcomes.

**Table 8:** Partial correlations between SLI children’s performance in the phonology, vocabulary and reading/spelling outcome measures and their filtered speech performance and rise time discrimination thresholds, controlling for age and IQ.

	Recognize LP	Recognize BP	Learn from LP	Learn from BP	Rise time threshold
Phonology (Oddity task)	**0.37^∗∗∗^**	**0.33^∗∗∗^**	0.09	-0.04	-0.20+
PSTM	0.21^∗^	**0.38^∗∗∗^**	0.09	0.01	-0.21^∗^
RAN	-0.20+	-0.15	-0.13	-0.21^∗^	0.25^∗^
BAS reading SS	0.20+	0.29^∗∗^	-0.08	-0.05	-0.27^∗∗^
BAS spelling SS	0.26^∗^	0.27^∗∗^	-0.13	-0.04	-0.21^∗^
BPVS SS	0.19^∗^	0.31^∗∗^	-0.14	-0.21^∗^	-0.18+
CELF receptive SS	0.20+	**0.45^∗∗∗^**	-0.08	-0.03	-0.16
CELF expressive SS	0.22^∗^	**0.40^∗∗∗^**	-0.12	-0.11	0.24^∗^

*+*p* < 0.10, ^∗^*p* < 0.05, ^∗∗^*p* < 0.01, ^∗∗∗^*p* < 0.001. LP, low pass filtered speech; BP, band pass filtered speech, PSTM, phonological short-term memory, RAN, rapid automatized naming, BAS, British Ability Scales; SS, standard score; BPVS, British Picture Vocabulary Scales. Uncorrected significance values; only the bolded values remain significant after applying the Benjamini and Hochberg (1995) correction for false discovery.*

### Discussion

For children with purely oral SLIs (‘classic’ SLI), the nursery rhyme recognition paradigm used here revealed significantly poorer recognition of band pass filtered speech targets compared to TD children, but not of low pass filtered speech targets. The sub-grouping of children with Pure SLIs and preserved IQ showed statistically equivalent performance to TD controls when recognizing low pass filtered speech (89 and 96% correct respectively in the first block of trials), and significantly poorer performance when recognizing band pass filtered speech (28 and 45% correct respectively). Indeed, both the SLI and TD groups showed higher accuracy with low pass filtered speech nursery rhymes than the older TD children tested in Experiment 1 (who recognized 78% of targets correctly in Block 1). This may reflect the fact that this younger cohort had more recent experience with nursery rhymes than the older children in Experiment 1 (nursery rhymes are part of the early school curriculum in the United Kingdom). Therefore, while all children found the band pass targets more difficult to recognize than the low pass targets, as also found for adults by Chait et al. (2015) and as also found in Experiment 1, in Experiment 2 these band pass filtered stimuli were *selectively more difficult* for the children with Pure SLI.

A notably different pattern of nursery rhyme recognition was observed for the children with SLIs and preserved IQ who also had phonological and reading difficulties. This SLI PPR sub-grouping showed statistically significant impairments compared to controls in recognizing *both* the low pass filtered speech stimuli and the band pass filtered speech stimuli. Therefore, the processing of slower temporal modulations in speech appears to be intimately related to the presence of phonological and reading difficulties (see also Fraser et al., 2010). Like the children with dyslexia tested in Experiment 1, who also had significant phonological and reading impairments, the SLI PPR children showed impaired performance with the slower temporal modulations that carry speech rhythm. Indeed, the SLI PPR children participating in the current study also showed significant impairments in perceiving rhythm patterns in both speech and music in a prior report, while the Pure SLI children did not (Cumming et al., 2015b). These theoretically interesting differences in performance for children with SLIs and children with developmental dyslexia are now discussed.

## General Discussion

Here we investigated in two independent studies the utility of a neural temporal sampling framework (Goswami, 2011) for explaining the etiology of developmental disorders of language learning. We proposed that while the AE rise time impairments that are found in children with dyslexia and SLIs may indicate a shared sensory difficulty in processing temporal modulation patterns in speech, the neural *temporal integration windows* that are most impaired may differ for each disorder. While temporal integration difficulties at slower timescales best characterize developmental dyslexia (Goswami, 2011, 2015), difficulties in temporal integration at rapid timescales may be a better characterization of children with SLIs (Tallal and Piercy, 1973). The novel filtered speech paradigm developed for studying temporal integration in adults by Chait et al. (2015) enabled independent assessment of our child participants’ ability to utilize slow versus faster temporal modulations for speech recognition. We investigated the perception of two kinds of filtered speech (low pass filtered and band pass filtered) by children with dyslexia in Experiment 1, and by children with SLIs in Experiment 2.

The data showed interesting differences in performance for children with phonological processing impairments (children with developmental dyslexia, and children with oral SLIs and phonological difficulties, SLI PPR) compared to children with oral SLIs only (non-phonological impairments in the comprehension and production of spoken language). In particular, the filtered speech paradigm revealed that children with dyslexia showed *impaired perceptual learning* in comparison to TD controls when listening to speech stimuli in which slow temporal modulations had been selectively extracted. Meanwhile, the SLI PPR children showed significant *speech recognition impairments* with low pass filtered speech, but no apparent perceptual learning difficulties. Both groups of children had preserved non-verbal IQ and equivalent years of experience of hearing spoken language to their TD controls, yet both groups showed a selective difficulty in processing temporal modulations at ∼4 Hz. However, the age difference between the two disorder groups meant that the SLI children did not also hear the nursery rhyme sentences in the “tricky alien” conditions, as this was judged to make the experimental procedures too long and demanding for our language-impaired participants. This difference in experimental method could potentially explain why the difficulties with low pass filtered stimuli showed up in perceptual learning for the older dyslexic children, and in speech recognition for the younger SLI PPR children.

By contrast, children with oral SLIs and no phonological processing difficulties (Pure SLI) showed selective recognition impairments for band pass filtered stimuli only, in which faster temporal modulations had been selectively extracted. Hence children with ‘classic’ SLI showed a selective difficulty in processing temporal modulations at ∼33 Hz, in the low gamma frequency range (30 – 50 Hz) typically characterized as the phonetic rate (Poeppel et al., 2008; Lehongre et al., 2011). The only grouping of children to show significant processing impairments at *both* modulation rates were the children with *both* oral SLIs and phonological processing difficulties (the SLI PPR group). This is suggestive of more severe impairments in temporal modulation processing in these children, that are not rate-specific. These data have implications for theoretical issues in the field of developmental language disorders, for the importance of slow versus faster temporal modulations in language development, and for the successful remediation of developmental dyslexia versus SLI.

Concerning theoretical issues, the data are supportive of Bishop and Snowling’s (2004) conclusions following their comprehensive literature review. Bishop and Snowling (2004) argued that classic developmental dyslexia and classic SLI were distinct disorders, and that the (often large) overlap found at the behavioral level in children with these two disorders of language learning did not necessarily mean that the disorders were *qualitatively* the same. Bishop and Snowling (2004) also observed an increasing trend in the developmental literature to group children with the two disorders together, with researchers testing single groups of “language learning impaired” children and reporting average performance (e.g., Kraus et al., 1996; Tallal, 2004). If the two disorders are in part aetiologically distinct, then studies that group such children together will be unable to identify significant causal factors.

With respect to the temporal sampling framework motivating the current study, it can be observed that both children with developmental dyslexia and children with SLIs showed impaired auditory sensory processing of AE rise time, theoretically related to identifying different temporal modulation patterns in speech (e.g., Corriveau et al., 2007; Goswami et al., 2011, 2013; Cumming et al., 2015a). Both groups of children tested here also showed impaired processing of syllable stress patterns and speech rhythm (see our previous reports: Goswami et al., 2013, dyslexia; Cumming et al., 2015a,b, SLI). As studies of normative infant populations across languages indicate that babies use speech rhythm and prosodic cues for encoding and parsing the continuous signal (Mehler et al., 1988; Echols, 1996), prosodic sensitivity is important for successful oral language acquisition (e.g., via ‘prosodic bootstrapping’, see Gleitman and Wanner, 1982). Indeed, recent modeling of the speech envelope of child-directed speech (English nursery rhymes) has shown that AMs nested in the envelope at key modulation rates (centered on ∼2 Hz, delta band; ∼5 Hz, theta band; ∼20 Hz, beta band) provide acoustic information relevant to the extraction of linguistic units, respectively stressed syllables, syllables, and onset-rime units (Leong and Goswami, 2015). Accordingly, auditory sensory (rise time) impairments that are present from birth could affect successful neural entrainment to the temporal modulation patterns in speech, affecting language acquisition from the “get-go” and impairing both language comprehension and production and phonological development.

In prior work, we have argued that both developmental dyslexia and SLI may reflect perceptual difficulties in processing slower temporal modulations in speech (<10 Hz, e.g., Goswami, 2011; Cumming et al., 2015a). The current study was able for the first time to compare speech recognition on the basis of faster versus slower temporal modulation patterns and to compare children with both disorders. The current data suggest that perceptual impairments with slower temporal modulations only characterize children with SLIs who also have phonological impairments (see also Fraser et al., 2010). Therefore, while the current data support the view that temporal sampling of modulations < 10 Hz is a primary impairment for children with developmental dyslexia, they do not support the view that temporal sampling of modulations < 10 Hz is a primary impairment for children with classic SLI. Rather, children with classic SLI appear to have selective difficulties with faster rate information. This is broadly consistent with proposals made originally about the sequential processing of rapidly-arriving brief acoustic cues by Tallal and Piercy (1973). More recently, Tallal has argued for a temporal integration deficit in windows of ∼40 ms (25 Hz) in children with SLIs (which she also called the ‘phonetic’ rate, see Tallal, 2004; see also Heim et al., 2011, for a potential link to oscillatory processes).

Concerning the importance of slow versus faster temporal modulations in language development, the literature is relatively sparse. Studies of oscillatory entrainment by infants show that even newborn infants entrain to amplitude-modulated noise at the contrasting rates of 3 Hz (∼ syllabic rate) and 40 Hz (∼ phonetic rate, see Telkemeyer et al., 2009, 2011). This suggests that both slower and faster temporal information is important from the beginning of language acquisition. For example, slower modulations may help with parsing, while faster modulations may help to specify native versus non-native phonemes. Furthermore, infants show right-lateralised responses for the slower temporal rates and bilateral responses for the faster temporal rates, consistent with a key tenet of multi-time resolution models of speech processing (Poeppel, 2003; Poeppel et al., 2008). This hemispheric asymmetry is also suggestive of *functionally independent neural networks* for the different temporal rates, which in principle could be impaired independently (as suggested by the data reported here). Meanwhile, our correlational and regression analyses showed that perceptual sensitivity to *both* slower and faster temporal modulations explained significant unique variance in the development of *both* spoken and written language skills. For developmental dyslexia, the strongest relations were found for spelling development (see **Table 4**), which is traditionally regarded as more intimately related to phonology than reading development. Recognizing low pass filtered speech accounted for 17% of unique variance in spelling after partialling out age and IQ, and recognizing band pass filtered speech accounted for 16% of unique variance in spelling. For SLI, the strongest relations were found for receptive and expressive language and recognition of band pass filtered speech. Recognition of band pass filtered speech accounted for 12% of unique variance in CELF receptive standard scores and 10% of unique variance in CELF expressive standard scores respectively. Meanwhile, recognition of low pass filtered speech accounted for 9% of unique variance in phonology. Overall, comparison of the two datasets is suggestive of equally critical roles for sensitivity to both slower and faster temporal information. Note that developmentally, impairments in processing slower versus faster temporal information in speech would be expected to lead to *different* patterns of neural compensation. This could be assessed in future studies.

Turning to the remediation of developmental language disorders, it is of interest that both children with developmental dyslexia and children with SLIs show impaired musical beat perception (Huss et al., 2011; Cumming et al., 2015b) and motor variability in synchronization to the beat (tapping to a rhythm, Thomson and Goswami, 2008; Corriveau and Goswami, 2009). Early language acquisition depends on multi-modal processing of speech information (auditory, visual and motor systems are involved in speech perception), hence these beat-related impairments could be suggestive with respect to musical remediation. Indeed, there is considerable interest in the wider field in the utility of musical therapies for both children with dyslexia and children with SLIs (e.g., Koelsch et al., 1999; Besson et al., 2007; Elmer et al., 2012). In earlier work, we have demonstrated that children with poor reading skills benefit from musical and rhythmic interventions focused around a beat rate of 2 Hz, in the oscillatory delta band (Bhide et al., 2013). By hypothesis, such interventions enable multi-modal sensory improvement of children’s processing of slower (rhythm-carrying) temporal modulations, thereby improving the accuracy of their neuronal rhythmic oscillatory entrainment to speech. We have suggested by contrast that musical interventions that support the extraction of *prosodic phrasing* in language may be of benefit for children with oral SLIs, rather than musical training based on simple beat-based rhythms (Cumming et al., 2015a,b). The data reported here suggest that our understanding of whether and when to offer prosodic-level training to both children with developmental dyslexia and children with SLIs (currently prosodic training is typically offered for neither disorder) requires further systematic and longitudinal study of children with classic SLI (children with oral language impairments but without phonological impairments). While the data reported here would support the use of musical and rhythmic interventions for remediating phonological processing difficulties for SLI PPR children, it is less clear that such interventions would benefit children with classic SLI.

Finally, given that behaviorally children with pure SLI do exhibit prosodic-level difficulties (Cumming et al., 2015a; Richards and Goswami, 2015), it would also be interesting to look in more detail at *learning trajectories* in the different developmental disorders. Again, longitudinal studies are required. For example, if younger children with SLIs or with developmental dyslexia had been tested here, they may also have shown difficulties in recognizing low pass filtered speech. Logically, it is also possible that processing difficulties in dyslexia associated with slower temporal modulations do not ameliorate with development (Hämäläinen et al., 2012), while similar processing difficulties in SLI do ameliorate with development. Different compensatory strategies may develop to support speech recognition in each disorder, with differential longitudinal effects. It is also theoretically important to discover the developmental time points at which these language learning disorders show maximum similarities in sensory processing and behavior. This information is critical with respect to selecting the most beneficial remediation at different developmental time points for affected children.

In conclusion, the novel filtered speech paradigm utilized here suggests that adopting a ‘temporal sampling’ framework for understanding developmental language disorders may support better understanding of both etiology and remediation. This will especially be the case if *cross-language* studies are conducted utilizing the TSF (Goswami, 2015). It is also timely to begin studying neuronal oscillatory entrainment to speech by children with SLIs, an enterprise that has already begun for TD children and for children with developmental dyslexia (Power et al., 2012, 2013). Given that there appears to be a shared difficulty in discriminating the temporal modulation patterns in speech in children diagnosed with the two language learning disorders, comparisons of neuronal entrainment at speech-relevant rates (delta, theta, beta, and low gamma) could reap rich rewards, both in terms of enhancing our understanding of the etiology of developmental disorders of language learning and in enhancing our ability to deliver more effective interventions to affected children.

## Author Contributions

UG conceived the experiments, analyzed the data and wrote the paper. RC helped program the task and tested the children. MC helped program the task. MH, NM, AW, and LB tested the children. TF took the lead on programming the task and tested the children

## Conflict of Interest Statement

The authors declare that the research was conducted in the absence of any commercial or financial relationships that could be construed as a potential conflict of interest.

The reviewer MB and handling Editor declared their shared affiliation, and the handling Editor states that the process nevertheless met the standards of a fair and objective review.
