# Supplementary material for: Perception of Filtered Speech by Children with Developmental Dyslexia and Children with Specific Language Impairments
Source: Front Psychol. 2016 May 30;7:791. doi: 10.3389/fpsyg.2016.00791 (PMC4885376; doi:10.3389/fpsyg.2016.00791)
Supplement: Supplementary file 1 [file Data_Sheet_1.DOCX]

Appendix. **Nursery rhymes used and schematic of experimental design**.

| **Nursery Rhyme** | **C/W** | **Envelope Filter** |
| --- | --- | --- |
| **Block 1** |  |  |
| Jingle jingle riddle car | W | 0-4Hz |
| incy wincy spider | C | 22-40Hz |
| check the till we need the bill | W | 0-4Hz |
| owl nacTonald has an arm | W | 0-4Hz |
| humpty dumpty sat on the wall | C | 0-4Hz |
| ten green bottles, sitting on the wall | C | 22-40Hz |
| jingle bells, jingle bells | C | 0-4Hz |
| hey diddle diddle, the cat and the fiddle | C | 22-40Hz |
| Have we curls gay and true | W | 22-40Hz |
| swing into the distance | W | 22-40Hz |
| **Block 2** |  |  |
| jumpy bumpy coming to call | W | 22-40Hz |
| a little riddle, to put in the middle | W | 0-4Hz |
| pendreen cockles, fitting in the hall | W | 0-4Hz |
| izzy whizzy glider | W | 0-4Hz |
| sing a song of sixpence | C | 0-4Hz |
| Happy Birthday to you | C | 0-4Hz |
| jack and jill went up the hill | C | 22-40Hz |
| single spells, single spells | W | 22-40Hz |
| old macdonald had a farm | C | 22-40Hz |
| Twinkle twinkle little star | C | 22-40Hz |
|  |  |  |
| **Block 3** |  |  |
| Jingle jingle riddle car | W | 22-40Hz |
| owl nacTonald has an arm | W | 22-40Hz |
| ten green bottles, sitting on the wall | C | 0-4Hz |
| jingle bells, jingle bells | C | 22-40Hz |
| swing into the distance | W | 0-4Hz |
| hey diddle diddle, the cat and the fiddle | C | 0-4Hz |
| check the till we need the bill | W | 22-40Hz |
| incy wincy spider | C | 0-4Hz |
| humpty dumpty sat on the wall | C | 22-40Hz |
| Have we curls gay and true | W | 0-4Hz |
| **Block 4** |  |  |
| sing a song of sixpence | C | 22-40Hz |
| single spells, single spells | W | 0-4Hz |
| jumpy bumpy coming to call | W | 0-4Hz |
| pendreen cockles, fitting in the hall | W | 22-40Hz |
| Happy Birthday to you | C | 22-40Hz |
| old macdonald had a farm | C | 0-4Hz |
| jack and jill went up the hill | C | 0-4Hz |
| izzy whizzy glider | W | 22-40Hz |
| Twinkle twinkle little star | C | 0-4Hz |
| a little riddle, to put in the middle | W | 22-40Hz |

Note. C = correct words, W = wrong words.
